# How the Statistics of Sequential Presentation Influence the Learning of Structure

**DOI:** 10.1371/journal.pone.0062276

**Published:** 2013-04-24

**Authors:** Devika Narain, Pascal Mamassian, Robert J. van Beers, Jeroen B. J. Smeets, Eli Brenner

**Affiliations:** 1 MOVE Research Institute Amsterdam, Faculty of Human Movement Sciences, VU University, Amsterdam, The Netherlands; 2 Laboratoire Psychologie de la perception, Université Paris Descartes, (CNRS UMR 8158), Paris, France; Bielefeld University, Germany

## Abstract

Recent work has shown that humans can learn or detect complex dependencies among variables. Even learning a simple dependency involves the identification of an underlying model and the learning of its parameters. This process represents learning a structured problem. We are interested in an empirical assessment of some of the factors that enable humans to learn such a dependency over time. More specifically, we look at how the statistics of the presentation of samples from a given structure influence learning. Participants engage in an experimental task where they are required to predict the timing of a target. At the outset, they are oblivious to the existence of a relationship between the position of a stimulus and the required temporal response to intercept it. Different groups of participants are either presented with a *Random Walk* where consecutive stimuli were correlated or with stimuli that were uncorrelated over time. We find that the structural relationship implicit in the task is only learned in the conditions where the stimuli are independently drawn. This leads us to believe that humans require rich and independent sampling to learn hidden structures among variables.

## Introduction

Structure learning is a class of Bayesian learning algorithms that was developed while studying unknown hierarchical dependencies among variables [1]–[6]. The learning of dependencies among variable classes is what we refer to here as structure learning. This framework has been proposed as an explanation for how the human brain may be able to generalize from very sparse observations [5], [7]–[12]. It has also been used to explain how the brain may be learning parameters at higher levels of the structural hierarchy to more efficiently resolve complex sensorimotor tasks [13]–[17]. And although much debate persists over whether the brain utilizes computation over such hierarchies as its modus operandi [18]–[19], there is evidence that humans learn hierarchical representations of parameters in sensorimotor tasks [14]. Theoretically, structure learning occurs at different levels of a hierarchical learning problem. In this work, we present humans with a hierarchical learning problem and focus on their learning of the highest level of structure in it, the model or relationship level. We are interested in how sequential presentation statistics influence the learning of structural relationships at hierarchical levels higher than those explored thus far in literature. We specifically look at how the statistics of sequential presentation of samples from a structure at such levels influences learning over time.

We simplify the structure of the learning problem by restricting it to two levels of analysis. One could visualize this problem as a graph with two hierarchical levels. The top level represents the class of ‘*models*’ (e.g. a linear relation) and the lower level represents the *parameters* of these relationships (e.g. slope and intercept of a linear relation). The brain does not know the real-world model and therefore may be entertaining multiple possibilities while trying to determine the actual nature of the relationship. For this reason, we shall refer to these possibilities as *hypotheses*. To perform the task effectively the brain would do well to identify the correct hypothesis and estimate the correct parameters. In this work, we empirically examine how the acquisition of the correct hypothesis and parameters is influenced by the sequential presentation statistics of samples from this structure.

To understand the particular problem we wish to investigate, let us consider a scenario where in an experiment, two naïve participants are given information about a certain property of a target and are asked to respond with their estimate of another property (of unknown value). In the initial stages, both participants appear to be at a loss to generate a correct response. As the experiment progresses, participant A is successful on frequent occasions whereas participant B appears to still be guessing. At the beginning of the experiment, the participants were unaware of a relationship between the two properties of the target. Participants could only discover the nature of such a relationship by careful observation of their errors over many trials. The correct identification of the nature of this relationship would enable participants to better predict an appropriate response for any observation. Let us assume in this case that the correct hypothesis is a linear relation between the observed variable and the response variable. Given that participant A is performing well, we could conclude that A has discovered the linear relationship inherent in the task whereas B has not. However, this may not necessarily be true. Unbeknownst to the participants, the experimenter has provided the two participants with an identical structure but two very different sequences of presentation to expose this structure. On every trial, participant A receives a target stimulus highly correlated with the previous stimulus, whereas participant B receives target stimuli that are independently sampled from a large range. Due to this manipulation, participant A receives stimuli that are closely related to each other and therefore the differences in required responses on consecutive trials are small. On the other hand it would be impossible for participant B to predict the appropriate response to a stimulus independently sampled from the structure without knowledge of the structure itself. Participant A, who is provided with a *Random Walk* pattern of stimuli over trials, is in a situation analogous to driving on a dark road for many hours with headlights illuminating a stretch ahead but having no notion of how the road curved in the last hour. Therefore it is possible to perform a task just by being able to predict the next immediate course of action but that may not necessarily lead to an understanding of global structure or form.

The question now arises, which of the two participants is more likely to identify the linear structural hypothesis as the correct choice. Participant B faces a sequence of uncorrelated stimuli, which makes predicting the required response almost impossible without an understanding of the structure. We believe that this scenario should induce a tendency to ‘explore’ possible structural hypotheses. Participant A may, on the other hand, ‘exploit’ an existing working strategy that allows them to perform the task but they may be unable to integrate long term information about the relationship and may therefore be less likely to select the correct hypothesis (linear). Conversely, if participants succeed in integrating long-term information, learning of the relationship during the *Random Walk* phase may be just as likely.

To examine which of these propositions is true, we performed an experiment such as the one described earlier with three groups of participants. All groups were presented with a linear relationship with identical parameters but the manner of sampling was different for each. One group was presented with a sequence of stimuli following a *Random Walk* where each stimulus was correlated with the previous one. The other two groups were presented with stimuli that were independently drawn from either a *Uniform* or *Gaussian* distribution. The higher probability of presentation of central stimuli for the *Gaussian* sequence results in better average predictability of a required response whereas it would be nearly impossible to predict future samples from within the range of the *Uniform* sequence. Further, each of the *Gaussian* stimuli sets was statistically matched (first and second order statistics) with its counterpart in the *Random Walk* group. It therefore serves as an intermediate condition between the *Uniform* and *Random Walk* groups.

In the *Random Walk* condition, merely correcting for the error incurred on the previous trial of correlated stimuli will produce a correlation in responses. This may not necessarily be indicative of actual learning of the structure. To ascertain whether learning truly occurs during the *Random Walk* presentation, we add a hundred uncorrelated trials at the end of the sequence. If participants learned the linear relationship, they should be able to generalize this learning to any distribution of samples. If they fail to do so, this would imply a failure to have learned the correct relationship inherent in the task.

## Methods

### Participants and Equipment

Twenty-four naïve participants (ten female), with normal or corrected-to-normal vision, provided written informed consent to perform the study. Participants were divided into three groups of eight participants. Additionally, seven naïve participants (three female) gave informed consent to perform a control experiment for one of the three conditions. The Ethical Committee of the Faculty of Human Movement Sciences, VU University approved the program to which this study belongs.

Participants were seated in the dark on a setup with their hands resting on a horizontal tablet surface (Wacom Digitizer UD-1825-A, 45.7×63.5 cm) upon which participants viewed computer generated images (refresh rate 85Hz, resolution: 1024×768 pixels) projected through a semi-silvered mirror (as described in [20]). Participants had no vision of their hand but obtained continuous veridical feedback about the position of their hand through the lit tip of the stylus they held.

### Stimulus and Task

**Figure 1:**
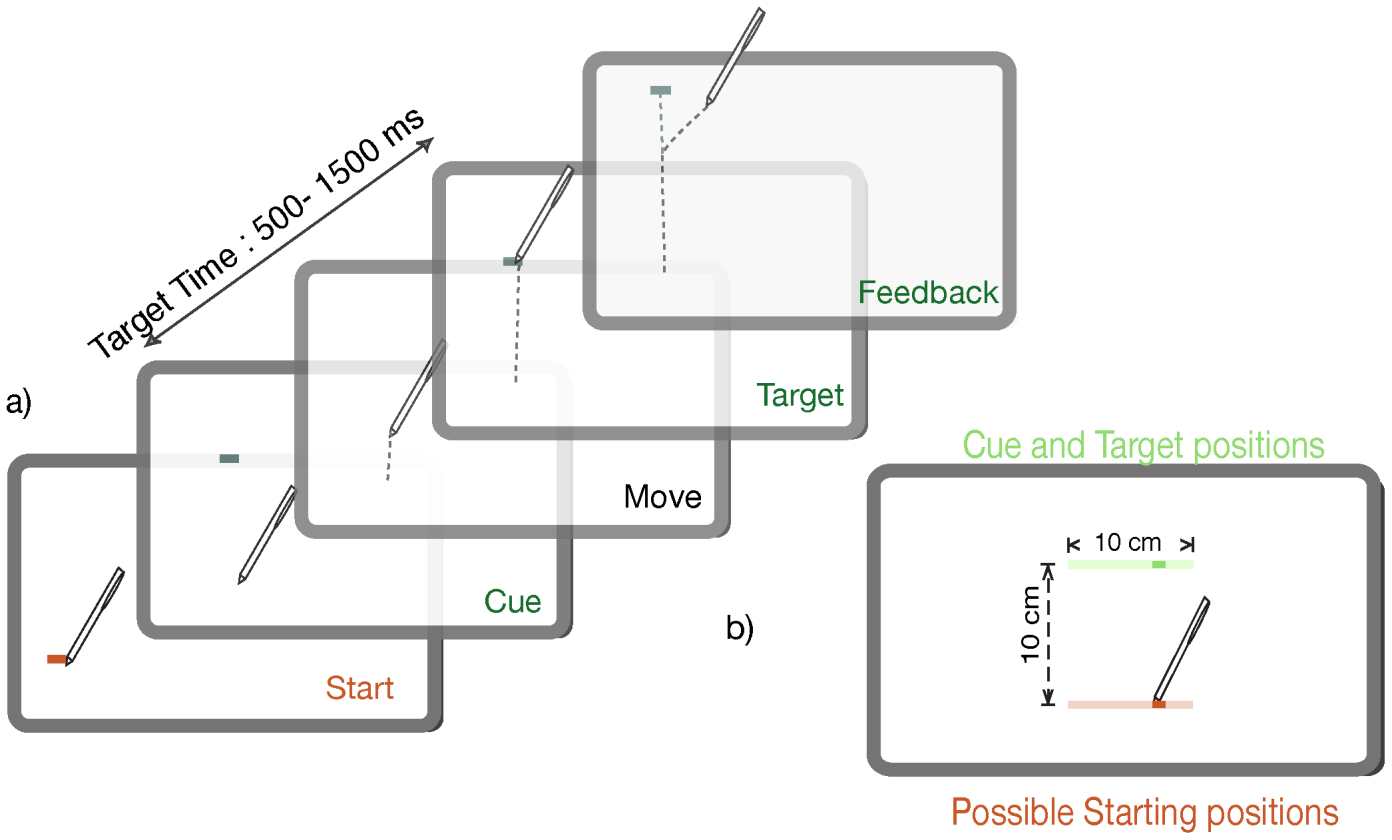
Trial timeline and design. a) Sequence of events in a single successful trial. Trials were initiated by moving the stylus to a starting location (red), which was followed by the flashing of a cue (green) and eventually the target. Hypothetical stylus trajectories are marked by dashed gray lines. Participants were required to anticipate the timing of the target. b) The spatial ranges of the target and starting position (shaded region) in the lateral dimension with an example pair (solid colors). The dimensions are marked by the dashed arrows (black).

Participants moved to a start location where after the lapse of a random interval (Uniformly sampled from a range of 500–2000 ms) the initiation of the trial was marked by the disappearance of the start location and the simultaneous flashing of a cue (on for 40 ms) at the future target location. The start location and the cue were always aligned for a given trial in their lateral coordinate but the cue appeared 100 mm further from the starting position in the sagittal direction (Figure 1 a). Participants were instructed beforehand that the target (2×5 mm) would appear at the cued location and they had to estimate when it would appear by intercepting it within 150 ms of its appearance after which time the target was extinguished. They were asked to move forward without reversing direction on each trial failing which they would forfeit the trial. Participants were naïve to the fact that the lateral spatial location of the target determined the time of its onset after the cue. The mapping between the target location (*x*) in millimeters and the onset time (*t*) in milliseconds was always as per the following equation: *t = *10*x* +1000. The lateral spatial location of the target ranged from −50 to 50 mm approximately (with respect to the center of the screen) and correspondingly the time of its appearance ranged from 500 ms to 1500 ms after the cue (Figure 1).

If the participant succeeded in intercepting the target, they saw the target flying upwards from the impact, received audio feedback, and received points. Participants had continuous veridical feedback from the lit tip of the stylus and could therefore see that they were too late or too early if they missed the target. Participants competed with each other for cumulative scores, which were made available to all participants.

### Design

**Figure 2:**
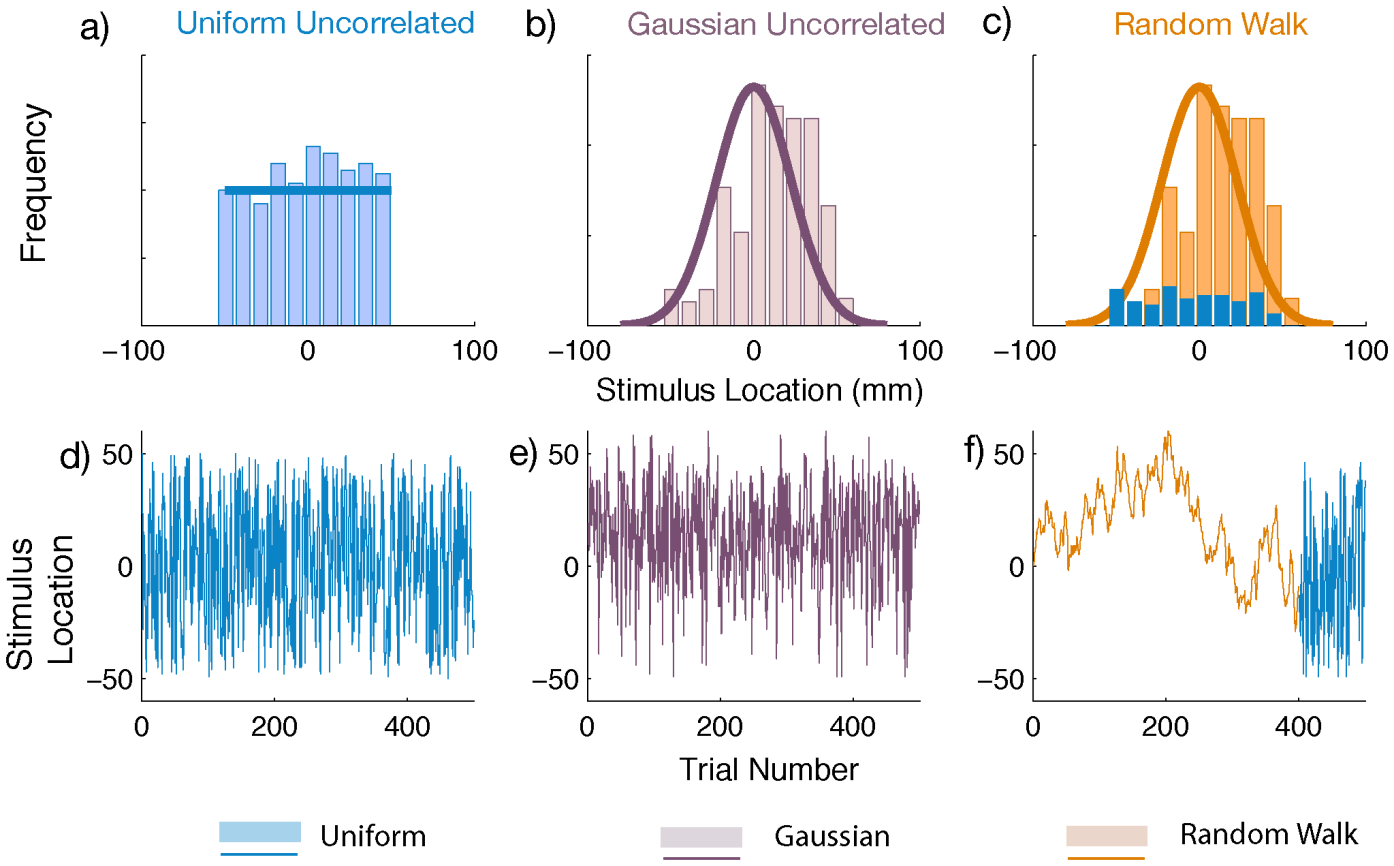
Distributions of the stimuli. a–c) Histograms (bars) of sample stimulus sets from each condition with average probability density fits (solid lines) to all sets. Note that the aggregate distribution of the Random Walk matches that of the Gaussian Uncorrelated condition. d–f) The corresponding sequences of presentation of the stimuli over the course of the experiment.

Each condition had 500 trials in total. For the *Random Walk* group, the stimulus location over trials took the form of a random walk selected such that without any temporal correlation, the sequence would closely resemble a Gaussian. They were also selected to approximately range within bounds of −50 to 50 mm, with a step noise drawn from a Gaussian distribution ( σ = 7 mm) (Figure 2c). Random walks were generated in mirrored pairs of 400 trials so that any systematic effects in the data resulting from a particular distribution would be cancelled out by its mirror. The last 100 trials in the *Random Walk* condition were drawn independently across trials from a *Uniform* distribution (Figure 2f). The *Gaussian Uncorrelated* stimuli sets were obtained by scrambling the Random Walk stimuli sets (Figure 2b). These were also produced in symmetric pairs since the Random Walks from which they were obtained were generated in reflected pairs, so that the mean position across pairs was zero.

The lateral locations of the *Uniform Uncorrelated* targets were drawn uniformly from the range : −50 to 50 mm (Figure 2d). We also scrambled 100 trials of the generated *Random Walk* into the *Uniform* distribution. Since large sequences of spatially constrained Random Walks are hard to produce, we selected some sequences where occasional targets drifted beyond the ±50 mm range.

### Data Analysis

We defined the response time as the time at which the participant’s stylus crossed the midline of the target. The spatial error was measured with respect to deviation from the center of the target. The 100 mm lateral stimulus space was divided into 5 bins of 20 mm each. For each participant, response times that exceeded thrice the standard deviation of response times within these bins were rejected (<1.5% of trials on average). Hardware delays were compensated online to calculate feedback (described in [20]). A hit was registered if the participant’s stylus was within the target region within 150 ms of its presentation (the display-time for the target).

Responses, over the course of the experiment, were grouped in 10 bins of 50 trials each. We performed robust regressions with a logistic weighting function (tuning factor = 1.2) within these bins. We calculated the regression slopes as proportions of the actual slope (10 ms/mm).

The last two bins of the *Random Walk* condition were actually uniformly distributed trials. For this reason, we performed a mixed design *Analysis of Variance* on the proportion slope regressed compared to the actual, with the condition as a between subjects factor and the first 8 bins of each condition as the within subjects factor. We also performed a similar ANOVA on the last two bins of each condition to analyze the learning at the end of each condition.

## Results

**Figure 3:**
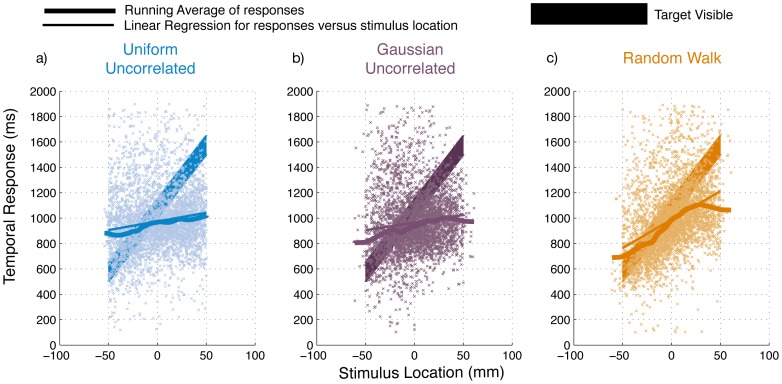
Temporal responses. Responses of all participants in each group are plotted against the stimulus location (mm) for the three groups: a) *Uniform Uncorrelated* b) *Gaussian Uncorrelated* and c) *Random Walk*. A running average of these pools is also displayed (thick lines) along with the linear regression (thin lines) performed on pooled subject data within each group.

Three groups of naïve participants performed an interception task in which a linear relationship was implicit. The mean movement initiation time was found to be 0.454 s (SD: 0.170 s). We found no significant differences in initiation times for the three groups (F_(2,21)_ = 0.38, p = 0.69 ). Figure 3 shows pooled responses for all participants within each group, running averages (window of 10 mm), and linear regression fits. For the *Uniform* group, the proportion of actual slope obtained by regressing the data is 18.5% of the actual slope (R^2^∶ 0.0213, F = 82, p<0.001), for the *Gaussian* it is 16.3% (R^2^∶ 0.014 F = 53.2 p<0.001 ) and it is 44.7% for the *Random Walk* (R^2^∶ 0.16 F = 724, p<0.001). Thus based on the regression obtained from the pooled data, the *Random Walk* participants have reproduced 45% of the slope provided in the stimuli whereas, the other two groups reproduce less than 20% of the actual slope. These values can be better evaluated in the light of the fact that even the most extensively trained author who had explicit knowledge of the parameters of the linear relationship was unable to obtain a slope percentage higher than 50% from their responses.

**Figure 4:**
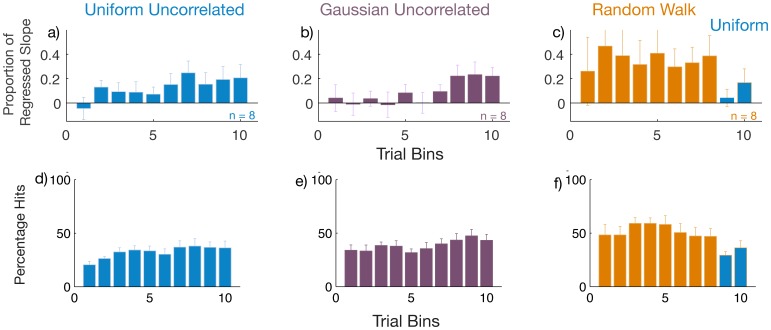
Learning and performance. The coefficient of robust regression as a proportion of actual slope in trial bins of 50 trials over the course of the experiment for the three conditions a) *Uniform Distribution* (blue), b) *Gaussian* (Purple) and c) *Random Walk* (orange). The last 2 bins in the *Random Walk* condition are uniformly distributed (blue). d, e, f: Percentage of hits within trial bins of 50 trials. Error bars indicate standard errors.

We divide the responses from each participant in bins of 50 trials throughout the experiment to obtain robust regression coefficients within these bins (Figure 4). We explained in earlier sections that any participant performing the task in the *Random Walk* group would incur a high correlation merely by responding with an adjustment to the error they incurred on the previous trial. Thus, to distinguish whether the overall high regression slope we obtained in Figure 3c is merely a consequence of following the *Random Walk* stimuli or arises from acquiring knowledge of the structure, we examine participants’ behavior when presented with the uniformly distributed trials after 400 *Random Walk* trials (Figure 4c). If the high slope obtained during the *Random Walk* bins is a consequence of learning the structure, the high correlation found earlier should continue despite the switch to the *Uniform* distribution trials. In Figure 4 c, we find that when the switch occurs at the 9^th^ bin (trial number 400), the regressed slope is statistically indistinguishable from zero (t_7_ = 0.59, p = 0.56). There is also a significant difference (t_7_ = 2.08, p = 0.036) between the regression slopes in the 8^th^ bin (last bin of the *Random Walk* in Figure 4c) and the ninth bin (first bin of the Uniform in Figure 4c). These findings indicate that participants in this group did not learn the structure during the *Random Walk* presentation.

We also performed an analysis of variance (ANOVA) on the proportion of slopes obtained by analyzing the three groups of conditions between participants and the first eight bins within groups (Figure 4 a b c). We found a main effect of condition (F_(2,21)_ = 5.71, p = 0.01 ). The main effect of bins and the interactions were not significant in this analysis. Pair-wise post-hoc least-squares difference (LSD) analyses on the three conditions revealed that the robust regression coefficients from the *Uniform* and *Random Walk* conditions differ from each other (p = 0.017), as do the *Gaussian* and *Random Walk* conditions (p = 0.005). The coefficients for the *Uniform* and *Gaussian* conditions do not differ (p = 0.56). Thus, during the course of the *Random Walk*, participants exhibited higher slopes than in the other two conditions. We also performed a mixed design ANOVA upon the regression coefficients in the last two bins of 50 trials each for the three groups but found no significant effects.

Figure 4 (lower panel) shows how the average percentage of hits across subjects changes over the course of the experiment. These rates are relatively low. We see from this that the task was relatively difficult for participants throughout. The percentage of hits in the Uniform and Gaussian conditions are lower than in the *Random Walk* condition, except after the switch to uniformly distributed trials occurs towards the end (Figure 4f). This provides further confirmation of the fact that these participants did not learn the correlation during the Random Walk (Figure 4c).

We see another interesting paradox while noting what happens after the switch in Figure 4c and f. It appears that the learned slope percent drops to zero once the switch to the *Uniform* distribution is made but the hit rate remains almost at par with that during the *Random Walk.* To understand this, let us consider an example where the participant has hit several stimuli appearing around the 0 mm mark resulting in a series of responses timed around 1000 ms. Suddenly the stimuli start appearing at locations that seem far apart but the participant, who continues to respond at 1000 ms, may still succeed in hitting some targets that appear close to the center. Therefore, had the participant chosen a fixed time of response from within the presented range of the stimuli, they would be likely to obtain a hit percentage of 15% within a bin of uniformly distributed trials, even without any knowledge of the linear relationship. In such an event, even with 15% hits, their regressed slopes would remain zero. This demonstrates how the regressed slope proportion is a more sensitive measure of the learning of the linear relationship in comparison with a measure of performance given by the hit rate.

One could also argue that participants in the *Random Walk* group did not learn the relationship since there was no apparent benefit in doing so since the strategy of responding with the help of the previous observation had been effective in the first 400 trials. We ran a control experiment to test whether introducing the possibility of there being a benefit to exploring multiple strategies would alter the result we found for the *Random Walk* group.

**Figure 5:**
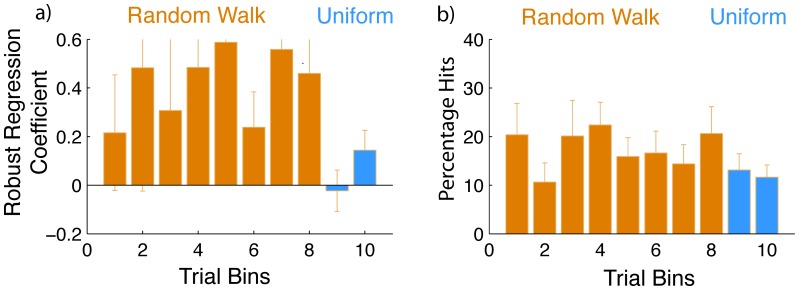
Results of control experiment. a) Average regression coefficients of participants who were informed of the switch from a *Random Walk to a Uniform* sequence (closely spaced and correlated vs. at random, during the last 100 trails). Axes are scaled to match earlier figures in the paper. b) Average of the Percentage Hits achieved in each bin. Error bars represent standard errors.

We asked seven naïve participants to perform the *Random Walk* condition precisely as described for the main experiment with one additional instruction. We gave participants prior information about the statistics of the sequence (*Random Walk* to *Uniform*) but gave them no indication of the nature of the linear relationship. Participants were told with emphasis that during the first 400 trials (trial numbers were displayed) stimuli would appear close to where they appeared on the previous trial but in the last 100 trials, the stimuli would appear at random locations on every trial. We found that the occurrence of the switch brought the percentage of learned slope to zero (Figure 5a), just as we had found in the main experiment (Figure 4c). The slope in bin 8 is significantly different from that in bin 9 (t_6_ = 2.65, p = 0.0378). This demonstrates that even with foreknowledge of the switch from the *Random Walk* to the *Uniform* distribution, participants failed to learn the linear relationship inherent in the stimuli.

## Discussion

We presented three groups of participants with the same linear relationship and parameters. Unbeknownst to the participants, this linear relationship existed between the location of the target and the required response time needed to hit it. We wanted to determine how participants differed in learning this relationship when the presentation sequence of stimuli was correlated over trials as opposed to when it was not. We expected that when the presentation sequence was uncorrelated over trials, participants would explore implicit relationships between variables in the task and therefore, would be more likely to learn the structure and select the correct hypothesis. On the other hand, when the stimuli were correlated, participants may have exploited an apparently adequate strategy (use of previous observation) and therefore may have never sought out a hidden dependency among variables in the task.

Our results support this hypothesis. We found evidence of detection of the correct linear hypothesis in the two conditions where no correlation existed over trials (Figure 4a & 4b) and could find no evidence of learning in the condition where samples were correlated over trials (*Random Walk:* Figure 4c). At a first glance (Figure 3c), it would seem that participants of the *Random Walk* group learned the correct hypothesis, however, the high slope obtained appears to be a mere consequence of passive tracking of correlated stimuli. Structure learning is a global phenomenon such that the observer has fully identified the hierarchies and dependencies involved. It therefore follows that if participants in the *Random Walk* condition had truly learned the structure, they should have easily been able to transfer such generic knowledge to any other distribution. This was, however, not the case. When participants in the *Random Walk* group switched to the uniformly distributed stimuli, their regressed linear slope dropped to zero suggesting the lack of a global realization of the structure. One could also argue that these participants may have failed to learn the structure because they used a strategy suited to perform the task optimally during the *Random Walk* sequence and continued to use it when they switched to uniformly distributed stimuli. And since the existing strategy was clearly yielding good predictions of future stimuli, there was no real requirement for participants to explore additional possibilities. This begs the question however, that if they were forewarned about it being useful to formulate multiple strategies, would they have performed any better. We found that they did not perform any differently in a control experiment using the Random Walk condition where participants were clearly instructed about the nature and timing of the switch (Figure 5). Participants did not alter their strategy to one that was better suited to stimuli experienced after the switch. This brings us back to what we argued earlier in the introduction that if a certain strategy yields satisfactory results, it may reduce the tendency of the observer to explore for more complex and globally conducive strategies.

In summary, the exploration of hierarchical dependencies implicit in an environment is facilitated by a rich and independent sampling of sequences. The *Random Walk* condition in the study reveals a paradox about participants being able to adequately perform a task without developing a global understanding of implicit relationships, like in the driving example in the introduction. Participants in the conditions where no correlation existed across trials, despite poorer performance, discover the structure inherent in the task. We may therefore conclude that there are certain tacit benefits to making errors.
